# The relationship between maternal smartphone use, physiological responses, and gaze patterns during breastfeeding and face-to-face interactions with infant

**DOI:** 10.1371/journal.pone.0257956

**Published:** 2021-10-08

**Authors:** Lilach Graff Nomkin, Ilanit Gordon

**Affiliations:** 1 Department of Psychology, Bar-Ilan University, Ramat-Gan, Israel; 2 Gonda Multidisciplinary Brain Research Center, Bar-Ilan University, Ramat-Gan, Israel; University of Iowa, UNITED STATES

## Abstract

Smartphone use during parent-child interactions is highly prevalent, however, there is a lack of scientific knowledge on how smartphone use during breastfeeding or face-to-face interactions may modulate mothers’ attentive responsiveness towards the infant as well as maternal physiological arousal. In the present study, we provide the first evidence for the influence of the smartphone on maternal physiological responses and her attention towards the infant during breastfeeding and face-to-face interactions. Twenty breastfeeding mothers and their infants participated in this lab study during which electrodermal activity, cardiograph impedance, and gaze patterns were monitored in breastfeeding and face-to-face interactions with three conditions manipulating the level of maternal smartphone involvement. We report that mothers’ gaze toward their infants decreased when breastfeeding while using the smartphone compared to face-to-face interaction. Further, we show that greater maternal electrodermal activity and cardiac output were related to longer maternal gaze fixation toward the smartphone during breastfeeding. Finally, results indicate that mothers’ smartphone addiction levels were negatively correlated with electrodermal activity during breastfeeding. This study provides an initial basis for much required further research that will explore the influence of smartphone use on maternal biobehavioral responses in this digital age and the consequences for infant cognitive, emotional, and social development.

## Introduction

The smartphone has become a dominant competitor for attention in our daily lives [1–3], potentially disrupting maternal sensitivity and attentive responsivity during mother-child interactions [4–11], especially in the critical infancy period [12] when the foundations of social interactions begin to form [13]. Despite the smartphone’s prevalence in everyday situations, little is known regarding its impact on maternal responsivity during early-life interactions with the infant. Responsive maternal behavior is an essential building block of the biobehavioral synchrony system that develops early on in life between mother and infant and promotes infants’ physiological, cognitive, and social-emotional growth [14]. This non-verbal system includes dynamical temporal concordance between mother’s and infant’s patterns of affect, proximity, touch, vocalizations and gaze patterns [14–17]. At around three months of age, infants engage in concurrent mutual gaze with their mothers approximately 30–50% of the time in face-to-face interactions [14]. Further, nearly every time infants looked at their mothers’ faces, mothers were already looking at them [18]. Overall, responsive and attentive parenting is known to have beneficial effects for children’s brain development [19, 20], self-regulation capabilities [21], and cognitive development [22].

In recent years, several studies have suggested, that smartphone use by the mother during interactions with her infant cause unpredictable distractions and might hinder her ability to sensitively respond to the infant and thus negatively influences resilience to stress and memory capacities in her child [23–26]. Therefore, it is critically important to explore the decrease in mothers’ attention to her infant cues [27] due to smartphone use, especially early in life in the primary interactive contexts of breastfeeding and face-to-face interactions. At three to six months of age, breastfeeding and face-to-face interactions comprise a majority of the settings in which the parent-infant bond develops and require sensitive responsivity from the mother [28–33] in order to continuously learn to recognize and interpret their infants’ cues and adapt to their changing abilities [34]. Therefore, the sensitive and responsive role of the parent in these fundamental parent-infant social contexts of feeding and face-to-face interactions is pivotal for early development.

As noted, a main competitor for maternal sensitive attention during interactions with her infant is the smartphone. Smartphone use at mealtime, for instance, is a common phenomenon among adults [7], and the presence of and engagement with screen distractions during infant feeding is one potential barrier to responsive feeding interactions [35]. Reports from an observational study in fast-food restaurants showed that caregivers who were focused on their mobile device during the meal, tended to respond to children’s bids for attention in insensitive or aggressive ways [9]. Family members of individuals with highly frequent mobile use in their home environment reported negative feelings such as frustration and stress [8]. Negative emotions related to smartphone use also accompanied parents’ attempts to minimize their phone use while children were in their care [6]. Moreover, these effects of smartphone use on the parent’s sensitive and attentive response towards their children were also found to be related to children’s risky behaviors [4] and to deficits in language acquisition in early childhood [10, 11]. To sum, parental distraction by smartphone use is a prevalent phenomenon that affects the quantity and quality of parents’ sensitive responses towards their children, and in turn, on their children’s behavior and affective responses.

The mere presence of the smartphone that has become a dominant competitor for human attention creating a cognitive distraction for adults as was shown with regards to working memory capacity, functional fluid intelligence [36], attentional and cognitive processes [37], and higher positive urgency [38].

Problematic excessive smartphone use is accompanied by stress like symptoms [39] that are reminiscent of substance-related dependence, such as withdrawal while not using the phone, and associated functional impairment [40–43]. The following factors have been shown to impact individuals’ compulsive use of their smartphones: their level of perceived enjoyment and satisfaction from smartphone use and its technological innovativeness, and their level of internet addiction [44, 45]. Smartphone addiction is a complex concept that includes various behavioral manifestations (e.g., gaming, chatting, shopping, and gambling) [46]. With respect to gender differences, higher levels of smartphone addiction were reported in women [46]. Although several studies have examined the negative impact of smartphone addiction [47–54], little is known regarding its behavioral and physiological basis, especially in the context of mother-infant interactions. A major way in which we can assess reactivity to smartphone distraction during mother-infant interaction is by measuring changes in physiological function of mothers’ peripheral nervous system as well as her gaze patterns during interactions with her infant that also involve the smartphone.

The sympathetic branch of the autonomic nervous system (SNS) is broadly related to high arousal states and to "fight or flight" responses [55]. SNS activity can be assessed via recordings of individuals’ impedance cardiography [56] and electrodermal activity (EDA) [55]. Impedance cardiography offers a non-invasive cardiac measurement in psychophysiological contexts such as cardiac output (CO) which describes the volume of blood being pumped by the left and right ventricle of the heart, per time unit [56]. Specifically, an increase in CO was detected while experiencing certain emotions such as anger [57], as well as in stress conditions [58, 59]. EDA is the continuous variation in the electrical characteristics of the skin, which can be measured directly as changes in skin conductance levels [55, 60, 61]. EDA has been shown to indicate psychophysiological arousal and to be a meaningful marker of psychologically significant stimuli [62], reactive in the contexts of addictions [63], mother-infant interaction and infants’ distress [64] as well as gaze behavior [61]. For these reasons, we expect EDA and CO to be good potential candidates for assessing the physiological effect of smartphone use during mother-child interactions.

### Aim and hypothesis

In light of the above, the aim of the current study was to explore the effects of smartphone use and smartphone distractions on maternal attention and physiological function during two types of interactions with her infant: breastfeeding and face-to-face interaction. First, in line with previous studies, we hypothesized that the mother’s attentiveness, indexed by her gaze direction toward the infant, will be reduced during moments of smartphone use compared to smartphone unavailability. We expected this effect to be more pronounced during breastfeeding compared to face-to-face interactions–as breastfeeding is a setting in which mothers’ can more easily disengage attention to their infants. As this is the first study to assess changes in EDA and CO during breastfeeding and face-to-face interactions with regards to the smartphone, we aimed to explore if physiological function in these indices will be different during the smartphone use versus smartphone unavailability stages and in breastfeeding versus face-to-face interactions. Finally, in line with previous studies, we hypothesized that higher maternal attention towards the smartphone will be related to a blunted maternal physiological arousal (similar to an addiction like state), as well as to increased reported smartphone addiction.

## Methods

The study’s protocol was approved by the Psychology Department’s Ethics Committee at Bar-Ilan University, Israel. Participants were informed that the purpose of the experiment was to examine maternal physiological processes during breastfeeding and how they relate to using a smartphone. All mothers provided informed consent at the onset of the study and all procedures are in line with the ethical approval obtained.

### Participants

Twenty mothers and their 3–6 months old infants (8 boys; 12 girls—65% were firstborns, 30% were born second, and 5% were born third) took part in the experiment. Criteria for study participation were mothers’ normal and corrected vision by contact lenses, and infant age was limited to three to six months. None of the mothers reported smoking cigarettes. Table 1 presents demographic information.

**Table 1:** Demographic characteristics of participants.

Demographic characteristics	
Mean age	30.2 (3.85)
mean age of infant (months postpartum)	5.05 (0.72)
Percentage reporting academic education	85
Percentage reporting above and average national wage	45
Percentage reporting receiving help with infant care	65

*Note*: Standard deviations are in parentheses.

Missing Data. One mother’s data could not be collected for technical reasons, therfore, she was not included in the analyses. In three other cases, physiological data could not be fully collected during the study because the infant was crying/the mothers needed to discontinue breastfeeding, or due to electrode displacement. During breastfeeding, under the conditions of smartphone use/bag, physiological data were obtained in full from 19 participants. During the mute condition, physiological data were obtained in full from 16 mothers. During the face-to-face interactions, while the smartphone was on mute, data was collected fully from 18 participants. A total of 16 had full data for the entire study.

### Procedure and experimental design

Before arriving at the lab, mothers completed several online questionnaires at home regarding their smartphone use, perceptions of their bond with the infant, ratings of their infant’s temperament, and demographic information. Upon arrival at the lab, mothers reported their baseline situational anxiety via a short questionnaire [65].

Participants were instructed to arrive at the lab session well hydrated and avoid caffeine/cigarettes two hours prior to the study. Mothers were physiologically monitored via a MindWare Mobile recording device (MindWare Technologies, Gahanna, OH) (See details on physiological collection and analysis below). Infants were not physiologically monitored during the study. After participants were connected to the physiological monitoring system, a five minutes baseline commenced, in which mothers were instructed to sit still and relax while their infant was under the care of a research assistant in a separate adjacent room. Following the baseline, mobile eye-tracking glasses were fitted to the mothers in order to measure their gaze patterns throughout all following experimental conditions. The entire lab visit was videotaped from three angles allowing full visibility of both interacting mother and infant. Video recordings, physiological recording, and SMI eye-tracking glasses were fully synchronized in time.

**Fig 1:**
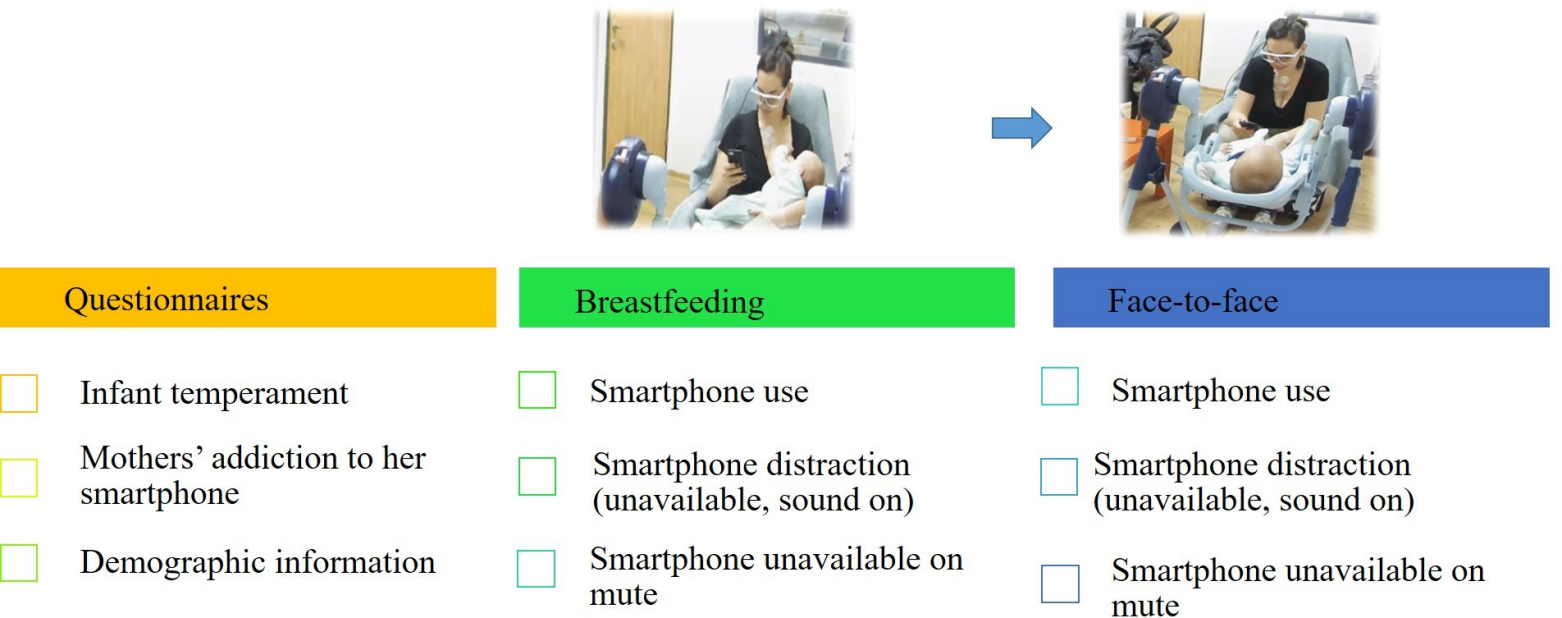
Procedure and experimental design. Initially, mothers’ filled in several self-report questionnaires, followed by two experimental phases in the lab (breastfeeding and face-to-face interactions). Each phase was comprised of three experimental smartphone conditions, each lasting 5 minutes. Through the lab study, mothers’ EDA, cardiac impedance and gaze fixation patterns were continuously recorded. The entire lab visit was also video-recorded from three angles.

The experiment was comprised of three conditions in two phases. The phases were breastfeeding and face-to-face interaction, starting with breastfeeding. The conditions were: 1) Smartphone use 2) Smartphone sound on but unavailable to mothers 3) Smartphone on mute and unavailable to mothers. The experiment started with participants being asked to place their smartphone (sound on) on the table next to them and with the assistance of the experimenter, to wear the eye-tracking glasses. Next, participants were instructed to take their baby in their arms and engage in breastfeeding for five minutes. During this condition, five WhatsApp messages were sent to the participants’ smartphone by the experimenter (sitting in an adjacent control room). Some text messages with questions that mothers were required to answer (e.g., "what brought you to participate in our study?") and some messages with further instructions for the following conditions as well as a short situational anxiety questionnaire identical to the ones completed at the arrival to the lab ("well done! Now I would like you to answer the questionnaire attached in the following link"). In the next condition (breastfeeding while smartphone in the bag), participants were asked to place their phone in their bag, sound on, and were instructed not to respond to any received alerts (the experimenter entered the room before the measurement began and assisted if needed) while breastfeeding for another five minutes. In this condition, four messages were sent by the experimenter, and the mothers’ only heard the notifications. In the final condition of the breastfeeding phase (breastfeeding/smartphone mute), mothers were asked to mute their phone and to place it back in their bags for another five minutes. Participants then were given the time to end breastfeeding, as they saw fit. The following phase of the face-to-face interaction was conducted similarly to the previous one, with the three phone conditions–each lasting five minutes. Mothers were asked to have a face-to-face interaction with their infants as they normally do ("you can use your baby’s toy or the puppets in the room as well as one of the books"). The infants were placed in a baby swing across from the mothers, who were instructed to remain sitting during the measurement and to avoid standing or taking their baby out of the swing. WhatsApp messages were then sent to the participants’ smartphones in the same manner as in the breastfeeding phase. Before each condition ended, the participants were asked to complete the situational anxiety questionnaire that was sent again via a text link. At the end of the measurement of the conditions in which the phone was not available, research assistant entered the room and instructed them orally to answer the questionnaire in the link. After the final task, there was a final five-minute baseline recording, identical to the first. Finally, monitoring equipment was then removed. Participants were escorted to a control room where they were asked to watch specific video clips from the six different experiment condition*phases that were just recorded, and to answer some questions on their emotions when watching the clips–valence and arousal (the Affect Grid) [66] (Fig 1). Data from the Affect analysis is not reported here. Mothers and infants were thanked and compensated for their participation (received a coupon for a free coffee and an onesie for the baby).

### Physiological measurements

We assessed physiological measures of the sympathetic branch of the autonomic nervous system (SNS) in mothers during breastfeeding and face-to-face interaction while also using their phone or getting notifications without being able to answer. We compared these conditions to a state in which the phone was muted and unavailable during interactions, so it could not overtly distract mothers. More specifically, we measured two types of physiological activity denoting SNS function: 1) Measures of electrodermal activity (EDA), assessed from the skin of the palm. 2) Measures of cardiological impedance, assessed from the heart (See details below on acquisition). We calculated statistics for two specific physiological measures of EDA: Tonic skin conductance level (SCL) and tonic period, and for one specific measure of cardiological impedance: Cardiac output (CO). Tonic SCL represents the tonic level of electrical conductivity of the skin calculated by the entire amount of SCL minus the phasic activity. Tonic period represents the amount of time spent in non-responsive states and is calculated by the total period of the waveform after removing all phasic responses (SCRs). Cardiac output indicates how much blood is being pumped out of the heart at any given time in liters per minute and is calculated as Heart Rate (HR) times Stroke Volume (SV).

#### Acquisition

Physiological data was obtained via a MindWare mobile impedance cardiograph device (MindWare Technology, United States) with a sampling rate of 500Hz. The device was connected to a participant via nine electrodes. Impedance and respiratory data was derived from the standard tetrapolar electrode procedure for the impedance cardiogram [67]. Electrodermal activity (measured in microsiemens [μS]) was collected via two disposable Ag-AgCl electrodes, both placed on the palm of the participant’s nondominant hand. EDA values were outputted from the MindWare EDA analysis software as the mean skin conductance level recorded at 2Hz.

### Gaze direction measurement

Maternal attention to the infant was operationalized via the assessment of mothers’ gaze patterns with an eye-tracking mobile device (Senso Motoric Instruments; Teltow, Germany, SMI, https://www.smivision.com)—a non-invasive and robust system designed to be used as a common pair of glasses (weight 75g). The SMI head-mounted system includes two small cameras on the rim of the glasses capturing the eye movements of the wearer, and a front view camera that captures the participant’s line of sight. The range of eye-tracking is 80° horizontal, 60° vertical with a binocular 60Hz temporal resolution and up to 0.1° spatial resolution. This is combined with a recording from a 24-Hz front view camera with a field of view: 60° horizontal and 46° vertical. Before the acquisition, for each participant, a three-point online calibration was conducted, followed by a check of the calibration’s accuracy. The calibration procedure included three dots on the wall at a distance of approximately 1 meter from the participant. Mothers with corrected vision (glasses or contact lenses) were instructed to use contact lenses in order for SMI glasses to be used.

### Data analysis

#### Physiological pre-processing

Cardiac output (CO) data were analyzed using MindWare Technology’s IMP application, version 3.1.2. The calculation of cardiac output and stroke volume was determined by Kubicek method [68] using rho set to 135 ohms. Artifacts in the data were detected by manual visual inspection with the MAD/ MED and IBI min/max heart beat detection methods that enabled noise filters with expected heart rate range of maximum Heart Rate (BPM) of 200 and minimum Heart Rate (BPM) of 40. The EDA signal was analyzed using MindWare Technology’s EDA software, version 3.1.4. Artifacts in the data were detected by manual visual inspection. Irregular pronounced changes and sudden drops and fluxes that were clearly related to disconnections, were corrected by a linear spline function available in the application. The smoothed EDA signal was achieved by a rolling filter of 500 data points per block. the level of EDA (in MicroSimmens) was outputted with threshold of 0.05 for every 500 ms (as recommended by the provider). We calculated statistics for three physiological measures mentioned above—tonic SCL, tonic period, and CO.

#### Gaze direction pre-processing

Visual intake count (fixations), saccades, and blinks were outputted by standard SMI algorithms. Areas of interest (AOI) were coded using SMI BeGaze software. AOI included infant (face and body) and smartphone. Visual intake count locations, indicated by a marker on the video recorded by the front view camera, were manually mapped by a trained research assistant, indicating fixation-by-fixation. Eye-tracking analyses were conducted according to a similar procedure in prior research [69, 70]. We derived the Dwell time (%) parameter to measure the duration of time in which mothers’ gaze fixated on an AOI. The dwell time refers to a visit in an AOI, from entry to exit, calculated by the sum of durations from all fixations and saccades that hit the AOI. Normalized dwell time parameter (dwell time in ms. divided by AOI Coverage) was derived to evaluate the differences between the mothers’ gaze patterns towards their smartphone and infant through the experiment smartphone use condition in both phases.

### Questionnaires

The Smartphone Addiction Scale (SAS) was used to measure daily-life smartphone use (disturbance, positive anticipation, withdrawal, cyberspace-oriented relationship, overuse, and tolerance) [48]. The following surveys were collected but not included in the current report: The Infant Behavior Questionnaire–Revised Very Short Form (IBQ-R) measures infant’ temperament [71]; The Mobile Attachment Scale (MAS) [39]; The Maternal-to-Infant Bonding Scale (MIBS) [72]; The short form of the State-Trait Anxiety Inventory [65]; The Affect Grid [66].

## Results

### The relationship between experimental phases and maternal gaze patterns

**Fig 2:**
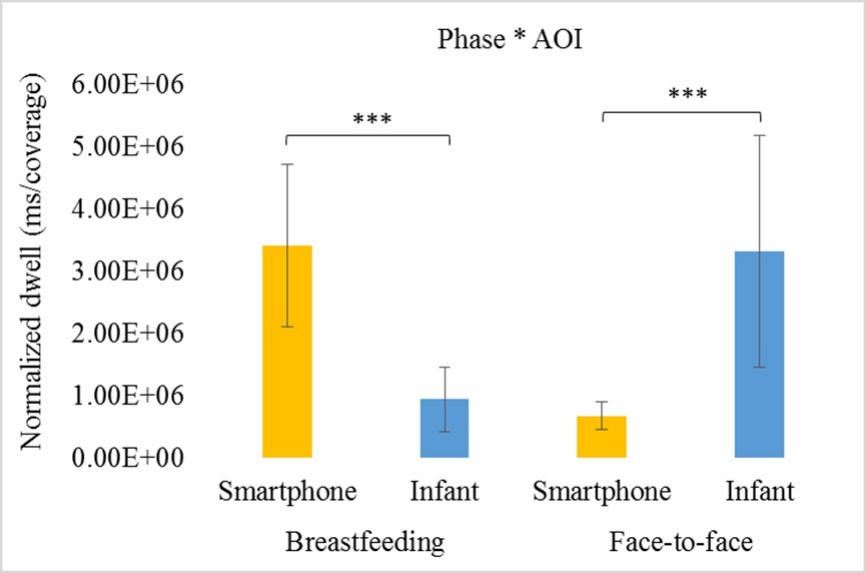
Means and SEs of normalized dwell towards infant or smartphone AOIs during the experimental phases. Normalized dwell refers to time differences measured in milliseconds in gaze patterns relative to the size of the AOI (ms/coverage). AOI = Area Of Interest (smartphone/infant). *** *p* < .001.

In order to assess if experimental phases and conditions, as well as maternal gaze direction (AOI: towards her infant or her phone), were related to the amount of maternal fixation on each AOI, we conducted a two-factor ANOVA with repeated measures (with Bonferroni post hoc correction for multiple comparisons). The analysis revealed a significant interaction effect between experimental phases (breastfeeding versus face-to-face) and AOIs in predicting the amount of the normalized dwell time (ms/coverage) mothers’ directed to each AOI [*F*(1,17) = 80.42, *p* < .001, *partial eta*^*2*^ = .83]. As can be seen in Fig 2, the normalized dwell time towards the smartphone was highest during breastfeeding (*M* = 3.41e+6, *SD* = 1.27e+6) and lowest during face-to-face interactions (*M* = 681,587, *SD* = 210,648). An opposite pattern was observed for the normalized dwell towards the infant–it was highest during face-to-face interactions (*M* = 3.19e+6, *SD* = 1.86e+6) and lowest during breastfeeding (*M* = 939,495, *SD* = 518,675) (Fig 2). No significant main effects were revealed for either the experimental phase [*F*(1,17) = .60, *p* = .45, *partial eta*^*2*^ = .03] or for the AOIs [*F*(1,17) = .01, *p* < .95, *partial eta*^*2*^ = .00].

### The relationship between experimental conditions, phases, and sympathetic responses

**Fig 3:**
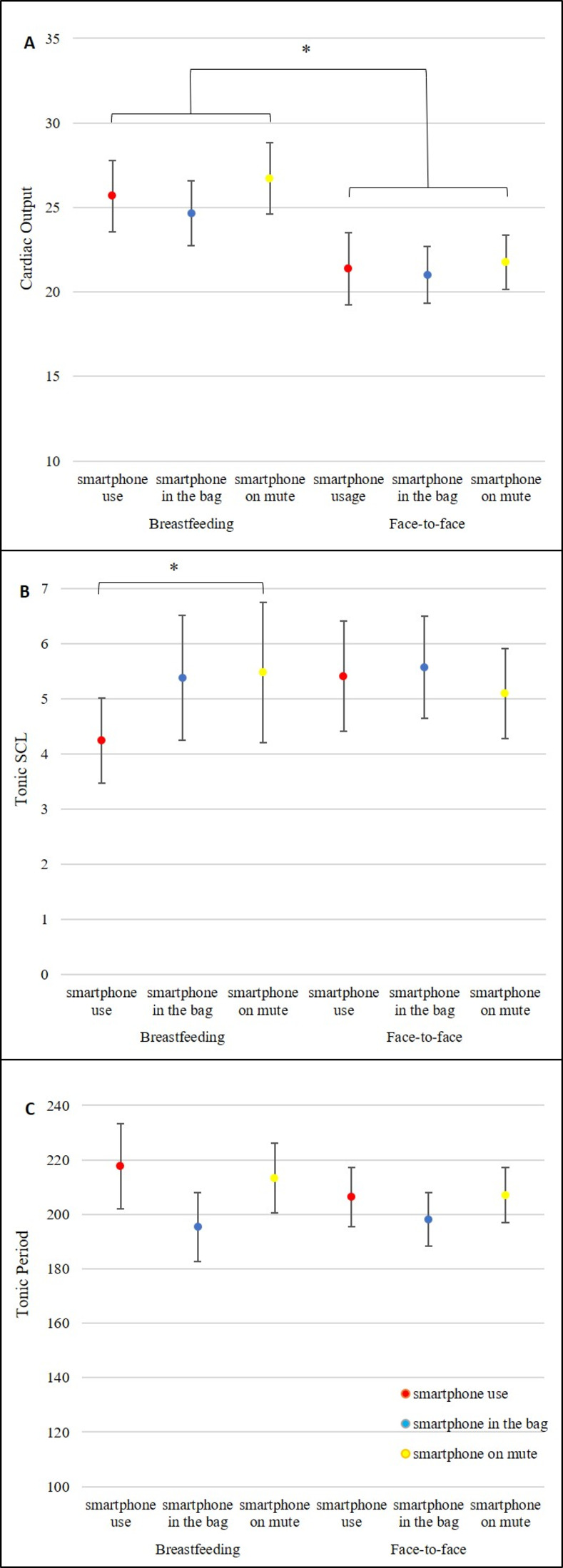
Changes in sympathetic responses throughout experiment conditions and phases: cardiac output (A), tonic SCL (B) and tonic period (C). Experimental phases: breastfeeding and face-to-face interaction. Experimental conditions (5 minutes each): Smartphone use, smartphone in bag (sound on) and smartphone on mute (in bag). **(A)** Cardiac output (CO; in liters per condition). Significance for experimental phases. * *p* < .05. **(B)** Tonic SCL = Tonic Skin Conductance Level, measured in microSiemens. Significance for the experimental conditions * *p* = .05. **(C)** Tonic period = time (in seconds) without skin conductance responses.

In order to assess if experimental conditions and phases were related to maternal physiological activity, we performed three repeated measures ANOVAs, in which CO, tonic SCL, and tonic period were the dependent variables. Independent variables were the experimental phases (breastfeeding and face-to-face interaction) and conditions (smartphone use, smartphone in the bag sound on, smartphone in the bag on mute). Results of the first ANOVA with CO as the dependent variable revealed no interaction effect between the experimental phases and conditions [*F*(2,26) = .23, *p* = .80, *partial eta*^*2*^ = .018]. A significant effect was found for the experimental phases [*F*(1,13) = 5.01, *p* = .04, *partial eta*^*2*^ = .28], such that CO was higher during breastfeeding (*M* = 26.60, *SD* = 2.10) compared to face-to-face interactions (*M* = 23.00, *SD* = 2.10). No effect was reveled for the experimental conditions [*F*(2,26) = 0.19, *p* = .83, *partial eta*^*2*^ = .01] (see Fig 3A).

Results of the second ANOVA with tonic SCL as the dependent variable revealed no interaction effect between conditions and phases [*F*(2,26) = .24, *p* = .79, *partial eta*^*2*^ = .018]. A significant effect was found for the experimental conditions [*F*(2,26) = 3.37, *p* = .05, *partial eta*^*2*^ = .21], such that tonic SCL was higher in the smartphone mute condition (*M* = 4.58, *SD* = 0.97) compared to the smartphone use condition (*M* = 3.80, *SD* = 0.97). No effect was found for experimental phases [*F*(1,13) = 0.38, *p* = .054, *partial eta*^*2*^ = .03] (see Fig 3B).

Results of the third ANOVA with tonic period as the dependent variable, revealed no interaction effect between conditions and phases [*F*(2,26) = .37, *p* = .70, *partial eta*^*2*^ = 0.03], no significant differences for maternal tonic period for the experimental conditions [*F*(2,26) = 2.290, *p* = .12, *partial eta*^*2*^ = .15] or for the experimental phases [*F*(1,13) = 0.18, *p* = .68, *partial eta*^*2*^ = .01] (See Fig 3C).

### Associations between smartphone addiction, physiological measures, and gaze patterns to the smartphone

In Table 2 we present Pearson’s correlations between self-reported smartphone addiction (SAS), physiological measures (tonic SCL, tonic period and CO), and maternal gaze fixations to her phone during breastfeeding.

**Table 2:** Correlation of the SAS, physiological measures and gaze towards the smartphone during breastfeeding and smartphone use.

		SAS		Normalized Dwell [ms/Coverage] to smartphone		Dwell time (%) to smartphone	
SAS		—		-.16		-.13	
EDA							
Tonic SCL		-.56	*	-.14		-.18	
Tonic Period		.32		.50	*	.56	*
ICG							
CO		-.10		.49	*	.57	*

Normalized dwell refers to time differences measured in milliseconds in gaze patterns relative to the size of the AOI (ms/coverage). AOI = Area Of Interest (smartphone/infant). SAS = Smartphone Addiction Scale. EDA = Electrodermal Activity. Tonic SCL = Tonic Skin Conductance Level. ICG = Impedance Cardiography. CO = Cardiac Output.

* *p* < .05.

### Associations between smartphone addiction and physiological indices

We used Pearson’s correlations to assess associations between the reported level of smartphone addiction and physiological measures. During breastfeeding: We found that tonic SCL during smartphone use and tonic SCL when the smartphone was on mute were negatively related to addiction scores (*r* = -.56, *p* = .01; *r* = -.58, *p* = .02 respectively). While in face-to-face interactions: We found that tonic SCL when the smartphone was in the bag and tonic SCL when the smartphone was on mute were negatively related to addiction scores (*r* = -.55, *p* = .01; *r* = -.50, *p* = .04 respectively). Additionally, during face-to-face interactions tonic period when the smartphone was in the bag was positively related to smartphone addiction scores (*r* = .50, *p* = .03). CO was not significantly related to addiction scores in any of the study’s phases or conditions (See all correlations in S1–S3 Tables). Overall the results indicate that in many of the study’s phases and conditions, higher EDA was related to lower levels of mothers’ addiction to their smartphones.

### Association between smartphone use and physiological indices–an initial exploration

**Fig 4:**
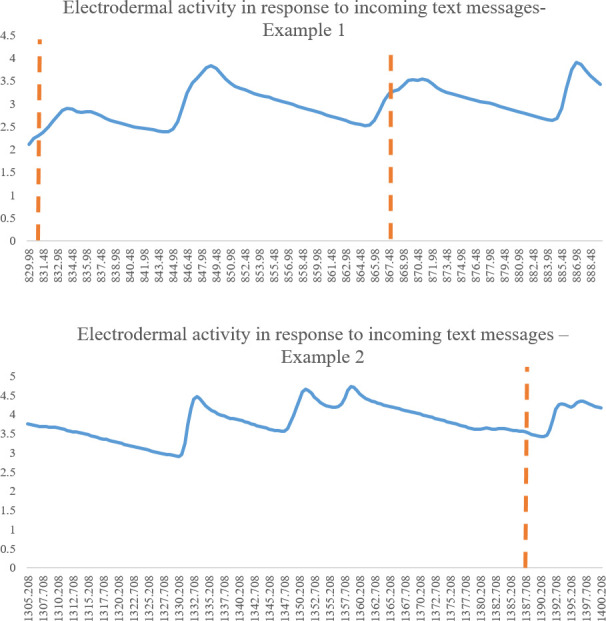
Two examples of changes in EDA during the smartphone use condition. The top and bottom panels depict examples of electrodermal activity (EDA) from two mothers who participated in the study during the smartphone use conditions. These figures illustrate skin conductance responses (SCRs). The red vertical dotted lines indicate an incoming text message, which was followed by the mother’s required shift of attention to her phone in order to respond. The Y axis represents electrodermal activity in microsiemens, and the X axis represents elapsed time. As can be seen, it seems, at least descriptively, that incoming messages can be followed by a distinctive skin conductance response.

In order to descriptively assess if there was a potential link between mothers’ shifting their gaze and attending to their smartphone and physiological arousal we looked at mothers’ skin conductance responses while getting notifications and having to read and respond to them. In Fig 4 we show two such examples from different mothers who participated in the study. As can be seen, at least descriptively, incoming messages (indicated by red vertical lines in the figures) were sometimes followed by a distinctive skin conductance response. These immediate responses may demonstrate that a shift in attention towards the smartphone was accompanied by a physiological arousal response (Fig 4).

## Discussion

Primary social interactions between mother and infant constitute early environmental conditions that are critical to infant development. However, in the last decade, the smartphone has emerged as a demanding competitor for parents’ attention. Despite the prevalence of this phenomenon, to date, there has been no scientific examination of how maternal smartphone use during breastfeeding or face-to-face interactions affects mothers physiological responses or their attention to their infants. In the present study, we examined mothers’ SNS activity and gaze patterns during breastfeeding and face-to-face interactions with their 3–6 months old infants, in which mothers were instructed to either use their smartphones, ignore them, or put them on mute. We further assessed the relationship between objective physiological markers and gaze patterns, and if they were related to subjective report of the mothers’ addiction to the smartphone.

As we hypothesized, during breastfeeding, mothers fixated on the smartphone for a longer duration than on their infant. An opposite maternal gaze pattern was found during face-to-face interactions: fixation towards the infants was longer compared to the smartphone. We suggest here that the breastfeeding context may be more susceptible to smartphone attentional distractions compared to the face-to-face context. Specifically, during breastfeeding, the infant is not demanding or available for face-to-face interaction, and the goal is to feed. As such, mothers may be more prone to distractions and other activities such as watching TV, thinking, or using their smartphones, compared to face-to-face interactions which require full attention to fulfill their goals [35].

We further found changes in maternal physiological activity that were related to the context of breastfeeding in the smartphone use condition. Specifically, only during breastfeeding, tonic SCL was elevated while the smartphone was on mute compared to when it was in use. This finding suggests that the smartphone’s unavailability during feeding interactions may be a potential cause of maternal alertness or distress, which is not the case during face-to-face interactions when the full attentiveness of the mother to the infant is called for. It is interesting to note that increased SCL was evident when the phone was unavailable and muted, but not when it was unavailable and the sound was on. It is possible that the muted condition induced a stress-like response due to a perceived lack of control [73], whereas the sound on condition promoted a sense of control that may have inhibited autonomic arousal [74].

Another main physiological finding, which was consistent across most of the phases/conditions in our study, indicates that mothers’ higher self-reported smartphone addiction scores correlated with lower EDA. These results on the physiological correlates of “chronic” smartphone use, echo results from the literature on substance addictions [75–77]. For instance, among individuals with extensive opioid use, there were little to no EDA changes following drug administration, while opioid-naïve or less experienced patients demonstrated more dramatic sympathetic changes after drug administration [78]. Additionally, heavy internet users showed lower SCL compared to low-risk internet users [63], and in gamblers, wins were related to hypoactive sympathetic functions [79]. It may very well be that due to extensive use, skin conductance is less responsive [78].

We further found that aspects of sympathetic activity were related to breastfeeding mothers’ gaze patterns towards the smartphone in a complex manner. Specifically, higher tonic period and higher CO were both related to longer maternal gaze fixation to her smartphone during breastfeeding. Tonic period represents the amount of time spent in non-responsive electrodermal states [80]. The mothers’ attention to the smartphone during breastfeeding can be considered as either related to the previously mentioned physiological hypoactivation [79], or may also represent a stress-reducing activity. Studies on the physiological response of contentment indicate decreased SCL [61, 81, 82], which fits to our results of less EDA responsivity related to more fixation on the smartphone during breastfeeding. High CO was also related to more fixation to the smartphone, which may potentially reflect an opposite stress-like response or higher arousal [83, 84]. Conversely, we suggest that this finding may reflect the fact that CO was overall higher during breastfeeding, in which there was also a higher level of maternal fixations to her phone. Our finding is also consistent with previous results from rodents [85] and humans [86], which pointed to an overall increase in CO during breastfeeding, possibly reflecting the perfusion of the mammary gland by oxytocin-infused blood, facilitating milk ejection. It is important to note that increased CO during breastfeeding may also be explained by the order of our paradigm’s phases, which was not counterbalanced but instead regularly started with breastfeeding to ensure the baby was calm. Therefore, further studies are needed to examine if elevated CO (and its relationship to fixation to the phone) is only due to breastfeeding or also interacts with smartphone use in a way we were not able to elucidate from the current design.

### Limitations and future studies

The limitations of the current study are mainly the small sample size and its exploratory nature, which entail that further studies corroborate and expand these initial results. Moreover, in the current study, we did not assess immediate physiological responses to received notifications, and hence, future studies are required to explore breastfeeding mothers’ immediate physiological responses when smartphone distractions occur. Further studies are also needed to examine if our results are unique to the breastfeeding context or may appear in other feeding situations in non-breastfeeding mothers. Another way in which our research design could be strengthened is to include a baseline condition in which the mother is not interacting with her infant but instructed to be involved with her smartphone “as she usually does”. This condition may reveal baseline differences in maternal proclivity towards checking her cell phone and could further develop the findings of the current study. An exploratory descriptive analysis of several mothers’ EDA time-series’ indicated there may be “incoming message”- specific physiological responses in EDA that accompany the shifts in attention from infant to smartphone. As we did not have a-priori hypotheses regarding these effects, we suggest they be further validated in future research. Finally, these results should be expanded in longitudinal research designs to examine the developmental influences of smartphone use on infants and the mother-infant dyad.

Notwithstanding our project’s strength is in its multimodal and interactive nature: A mental, behavioral, and physiological examination of mothers during real-life situations that involve interactions with their infant and smartphone use. To our knowledge, this is the first presentation of data and findings that characterize behavioral and physiological markers of a preference to the smartphone in mothers’ that is context specific—breastfeeding versus face-to-face interactions. Interestingly, some physiological responses were similar to those reported in the literature on substance addiction. Our results should be further explored to fully understand the outcomes of smartphone use on characteristics of the mother, the infant and their bond in this digital era.

## Supporting information

S1 Table Correlations between tonic SCL and smartphone addiction scores (SAS). Tonic SCL = Tonic Skin Conductance Level, measured in microSiemens. * *p* < .05.
(DOCX)

S2 Table Correlations between tonic period and smartphone addiction scores (SAS). * *p* < .05.
(DOCX)

S3 Table Correlations between CO and smartphone addiction scores (SAS). CO = Cardiac output (in liters per condition). * *p* < .05.
(DOCX)

S1 Data (XLSX)
