# Supplementary material for: The relationship between maternal smartphone use, physiological responses, and gaze patterns during breastfeeding and face-to-face interactions with infant
Source: PLoS One. 2021 Oct 8;16(10):e0257956. doi: 10.1371/journal.pone.0257956 (PMC8500426; doi:10.1371/journal.pone.0257956)
Supplement: S1 Table — Tonic SCL = Tonic Skin Conductance Level, measured in microSiemens. * p < .05. (DOCX) [file pone.0257956.s001.docx]

**S1 Table.** **Correlations between tonic SCL and smartphone addiction scores (SAS).**

| **SAS** |  | Breastfeeding | |  |  | Face-to-face |  |
| --- | --- | --- | --- | --- | --- | --- | --- |
| smartphone use | | N = 19 |  |  |  | N = 20 |  |
|  | Pearson's r | -0.56 | * |  |  | -0.38 |  |
|  | p-value | 0.01 |  |  |  | 0.09 |  |
| smartphone in bag | | N = 19 |  |  |  | N = 20 |  |
|  | Pearson's r | -0.42 |  |  |  | -0.55 | * |
|  | p-value | 0.07 |  |  |  | 0.01 |  |
| smartphone on mute | | N = 16 |  |  |  | N = 18 |  |
|  | Pearson's r | -0.58 | * |  |  | -0.50 | * |
|  | p-value | 0.02 |  |  |  | 0.04 |  |
| Note. * < .05 | | | |  |  |  |  |
