# Supplementary material for: The relationship between maternal smartphone use, physiological responses, and gaze patterns during breastfeeding and face-to-face interactions with infant
Source: PLoS One. 2021 Oct 8;16(10):e0257956. doi: 10.1371/journal.pone.0257956 (PMC8500426; doi:10.1371/journal.pone.0257956)
Supplement: S2 Table — * p < .05. (DOCX) [file pone.0257956.s002.docx]

**S2 Table. Correlations between tonic period and smartphone addiction scores (SAS).**

| **SAS** |  | Breastfeeding | |  |  | Face-to-face |  |
| --- | --- | --- | --- | --- | --- | --- | --- |
| smartphone use | | N = 19 |  |  |  | N = 20 |  |
|  | Pearson's r | 0.32 |  |  |  | 0.19 |  |
|  | p-value | 0.18 |  |  |  | 0.41 |  |
| smartphone in bag | | N = 19 |  |  |  | N = 20 |  |
|  | Pearson's r | 0.31 |  |  |  | 0.50 | * |
|  | p-value | 0.20 |  |  |  | 0.03 |  |
| smartphone on mute | | N = 16 |  |  |  | N = 18 |  |
|  | Pearson's r | 0.45 |  |  |  | 0.24 |  |
|  | p-value | 0.08 |  |  |  | 0.35 |  |
| Note. * < .05 | | | |  |  |  |  |
