# Supplementary material for: The relationship between maternal smartphone use, physiological responses, and gaze patterns during breastfeeding and face-to-face interactions with infant
Source: PLoS One. 2021 Oct 8;16(10):e0257956. doi: 10.1371/journal.pone.0257956 (PMC8500426; doi:10.1371/journal.pone.0257956)
Supplement: S3 Table — CO = Cardiac output (in liters per condition). * p < .05. (DOCX) [file pone.0257956.s003.docx]

**S3 Table. Correlations between CO and smartphone addiction scores (SAS).**

| **SAS** |  | Breastfeeding | |  |  | Face-to-face |  |
| --- | --- | --- | --- | --- | --- | --- | --- |
| smartphone use | | N = 19 |  |  |  | N = 20 |  |
|  | Pearson's r | -0.1 |  |  |  | -0.03 |  |
|  | p-value | 0.68 |  |  |  | 0.91 |  |
| smartphone in bag | | N = 19 |  |  |  | N = 20 |  |
|  | Pearson's r | -0.30 |  |  |  | -0.20 |  |
|  | p-value | 0.22 |  |  |  | 0.41 |  |
| smartphone on mute | | N = 16 |  |  |  | N = 18 |  |
|  | Pearson's r | 0.14 |  |  |  | -0.23 |  |
|  | p-value | 0.60 |  |  |  | 0.36 |  |
|  |  |  |  |  |  |  |  |
